# Antibacterial Activity of *Arbutus pavarii* Pamp against Methicillin-Resistant *Staphylococcus aureus* (MRSA) and UHPLC-MS/MS Profile of the Bioactive Fraction

**DOI:** 10.3390/plants9111539

**Published:** 2020-11-11

**Authors:** Nawal Buzgaia, Tahani Awin, Fakhri Elabbar, Khaled Abdusalam, Soo Yee Lee, Yaya Rukayadi, Faridah Abas, Khozirah Shaari

**Affiliations:** 1 Department of Chemistry, Faculty of Science, University of Benghazi, Benghazi, Libya; nawalbuz@gmail.com (N.B.); awin_org@yahoo.com (T.A.); fakhri.dr@gmail.com (F.E.); 2 Natural Medicines and Products Research Laboratory (NaturMeds), Institute of Bioscience, Universiti Putra Malaysia, UPM Serdang 43400, Selangor, Malaysia; khaledbashirala.79@gmail.com (K.A.); daphne.leesooyee@gmail.com (S.Y.L.); yaya_rukayadi@upm.edu.my (Y.R.); faridah_abas@upm.edu.my (F.A.); 3 Department of Microbiology, Faculty of Science, University of Gharyan, Gharyan, Libya; 4 Department of Food Science, Faculty of Food Science and Technology, Universiti Putra Malaysia, UPM Serdang 43400, Selangor, Malaysia; 5 Department of Chemistry, Faculty of Science, Universiti Putra Malaysia, 43400 UPM Serdang, Selangor, Malaysia

**Keywords:** *Arbutus pavarii*, antibacterial activity, MRSA, time–kill curves, ultrahigh-performance liquid chromatography, mass spectrometry (UHPLC- ESI-MS/MS)

## Abstract

*Arbutus pavarii* Pamp is a medicinal plant commonly used by local tribes in East Libya for the treatment of many diseases, such as gastritis, renal infections, cancer and kidney diseases. In this study, the antibacterial activity of the leaf and stem bark extracts of the plant against methicillin-resistant *Staphylococcus aureus* (MRSA), as well as the metabolite profiles of the bioactive fractions, was investigated. The antibacterial activity was determined by disc diffusion method, minimum inhibitory concentration (MIC) and minimum bactericidal concentration (MBC), while the microbial reduction by the bioactive fraction was evaluated using time–kill test. The bioactive fraction was further subjected to ultrahigh-performance liquid chromatography–mass spectrometry (UHPLC-ESI-MS/MS) analysis to putatively identify the chemical constituents contained therein. All the extracts and fractions showed different levels of antibacterial activity on the tested MRSA strains. The highest total antibacterial activity, i.e., 4007.6 mL/g, was exhibited by the crude leaf methanolic extract. However, the ethyl acetate fraction of the leaf showed moderate to significant antibacterial activity against MRSA at low MIC (0.08–1.25 mg/mL). Metabolite profiling of this fraction using UHPLC-ESI-MS/MS resulted in the putative identification of 28 compounds, which included phenolic acids, flavan-3-ols and flavonols. The results of this study showed that the ethyl acetate fraction of *Arbutus pavarii* leaf possessed potential antibacterial activity against MRSA and hence can be further explored for pharmaceutical applications as a natural antibacterial agent.

## 1. Introduction

Millions of people are affected by contagious bacterial diseases throughout the world. These infectious diseases have persistently caused disability and death throughout mankind’s history. According to the World Health Organization (WHO), approximately 50,000 people die from bacterial infectious diseases throughout the world every year [1]. Methicillin-resistant *Staphylococcus aureus* (MRSA) is a group of Gram-positive bacteria that are distinct from other strains of *Staphylococcus aureus* [2]. MRSA is usually found in hospitals, prisons and nursing homes, where the people with open wounds and deteriorated immune systems are at greater risk of hospital-acquired infections. Although MRSA began as a hospital-acquired infection, it can be found in all communities and livestock. The terms HA-MRSA (healthcare-associated or hospital-acquired MRSA), CA-MRSA (community-associated MRSA) and LA-MRSA (livestock-associated) reflect the MRSA infections in a variety of hosts [3]. The MRSA displayed resistance against many antibiotics such as methicillin, a semisynthetic β-lactam antibiotic. Generally, the β-lactam mechanisms of resistance of MRSA strains support cross-resistance to all β-lactam antibiotics [4]. The key mechanism for resistance is the enzyme-catalyzed modification and ultimate destruction of the antibiotic, causing its dynamic efflux from cells and antibiotic target alteration [5]. Therefore, there is a high demand to develop antibiotics from natural sources based on medical plant extracts in a bid to back up the effectiveness and potency of conventional antibiotics [6]. Natural products play an important role in drug discovery, as evidenced by over 50% of all modern clinical drugs being of natural product origin [7].

Medicinal plants are rich sources of secondary metabolites with various biological properties, including antimicrobial properties [6,8]. *Arbutus pavarii* Pamp, an endemic medicinal plant species known locally as Shmar in Libya, is an evergreen shrub belonging to the Ericaceae family [9,10]. In folk medicine, it is used for the treatment of gastritis, renal infections, cancer ailments and kidney diseases [11]. Previous phytochemical studies on *A. pavarii* showed that this plant contains mainly flavonoids, tannins, glycosides, simple phenolics, triterpenes and sterols [11]. In addition, it was also reported that *A. pavarii* demonstrated strong antibacterial activity against several pathogenic bacteria [11]. However, few studies have focused on determining the effect of *A. pavarii* extracts and its fractions against resistant bacterial strains. Thus, the aim of this study was to evaluate the *A. pavarii* leaf and stem bark extracts against methicillin-resistant *Staphylococcus aureus* (MRSA). The anti-MRSA activity of the crude methanolic extract and various solvent fractions were assayed using disc diffusion assay, followed by minimum inhibitory concentration (MIC) and minimum bactericidal concentration (MBC) determinations, as well as time–kill curve analysis. In addition, the active fraction was subjected to ultrahigh-performance liquid chromatography–mass spectrometry (UHPLC-ESI-MS/MS) analysis for the identification of potential bioactive compounds.

## 2. Results and Discussion

### 2.1. Antibacterial Activity Of A. pavarii Crude Extracts and Solvent Fractions

Disc diffusion test was used to first screen the crude methanolic extracts and solvent fractions for presence of antibacterial activity. The appearance of zones of inhibition produced around the discs was observed, and their diameters were measured and recorded (Table 1). The standard antibiotic, 0.1% CHX, showed inhibition zones ranging from 7.00 to 10.33 mm against the bacterial strains. At the test concentration of 10 mg/mL, the crude methanolic extracts of the leaf and stem bark showed inhibition zones in the ranges of 8.00–9.67 mm and 7.00–10.00 mm, respectively. Among the different solvent fractions of the leaf, the EtOAc fraction showed the greatest activity towards all the bacterial strains, giving inhibition zones of 13.66, 12.00, 13.67 and 13.00 mm against MRSA ATCC 700699, MRSA KCCM 12255, MRSA1 and MRSA2, respectively. The same trend was observed for the stem bark fractions. However, compared to the leaf EtOAc fraction, the stem bark EtOAc fraction showed smaller inhibition zones of 8.00–9.00 mm, indicating that the stem bark either contained different bioactive constituents or lower amounts of the same bioactive constituents [12]. Other solvent fractions of the leaf and stem bark showed no to weak activities against the test bacteria. Previously, Alsabri et al. [13] investigated the antibacterial properties of solvent extracts prepared from the aerial part of *A. pavarii*. They reported that the methanol extract exhibited the highest activity against *S. aureus*, *Escherichia coli* and *Candida albicans*. The chloroform extract was active only against *S. aureus*, while the n-hexane extract showed activity against *C. albicans*. Overall, these results indicated that the polarity of the solvent plays an important role in the extraction of the active ingredients and consequently in their potential antimicrobial activity.

The calculated relative inhibition zone diameter (RIZD) values of the test samples against the MRSA strains varied from 70.99% to 171.43%, as shown in Table 1. The RIZD value provides additional information showing the differential effects of the test extracts and fractions compared to the standard antibiotic used as a positive control. An RIZD value >100% means that the tested extract is more effective than the antibiotic. The leaf EtOAc fraction demonstrated the highest RIZD values, ranging from 125.85% to 171.43% against all MRSA strains. The higher RIZD percentages demonstrated by the leaf EtOAc fraction are a good indication that the leaf of *A. pavarii* contained most, and probably in higher amounts, of the antibacterial compounds of this plant species. It is worthy of note that the local population frequently uses the leaf material for medicinal purposes [11]. Based on the higher biological activity, the leaf and the stem bark EtOAc and n-BuOH fractions were subjected to further evaluation of their MIC, MBC and total activity values. 

### 2.2. Bacteriostatic (MIC) and Bactericidal (MBC) Effects of Bioactive Extracts and Fractions

The antibacterial activity of the bioactive extracts and fractions was further investigated through the determination of the MIC and MBC values, as well as the total activity. The MIC and MBC values are presented in Table 2. The MIC values of the crude leaf methanolic extracts ranged between 0.08 and 1.25 mg/mL, while the MBC values ranged between 0.16 and 2.50 mg/mL. The leaf methanolic extract was more potent against the two standard MRSA strains, i.e., ATCC 700699 (MIC 0.08 mg/mL; MBC 0.16 mg/mL) and KCCM 12255 (MIC 0.63 mg/mL; MBC 1.25 mg/mL), in comparison to the clinical isolates, against which it showed MIC of 1.25 mg/mL and MBC of 2.5 mg/mL for both strains. A similar trend of potency was observed for the leaf fractions, where the standard MRSA strains were more susceptible to the fractions while the clinical isolates were less affected. The MIC and MBC for the EtOAc fractions were in the ranges of 0.08–1.25 mg/mL and 0.16–2.50 mg/mL, respectively; the MIC and MBC for n-BuOH fractions were 0.04–2.50 mg/mL and 0.08–5.00 mg/mL, respectively. In addition, among the activities exhibited by the leaf extract and fractions on the MRSA strains, the n-BuOH fraction showed the highest potency against the ATCC 700699 strain with MIC and MBC values of 0.04 and 0.08 mg/mL, respectively. The antimicrobial activity of an extract is considered very interesting and is of significant scientific value when its MIC values are lower than 100 μg/mL [14]. Hence, the present results revealed that the *A. pavarii* leaf methanolic extract and solvent fractions have moderate to significant activity against the tested MRSA strains.

On the other hand, the MIC values of the crude stem bark methanolic extract were lower than the leaf extract, ranging between 0.63 and 1.25 mg/mL, while the MBC values ranged between 1.25 and 2.50 mg/mL. The stem bark methanolic extract was more potent against the standard MRSA strain, ATCC 700699 (MIC 0.63 mg/mL; MBC 1.25 mg/mL), than against MRSA KCCM 12255 and the two clinical isolates as it showed MIC and MBC values of 1.25 and 2.5 mg/mL, respectively, against these three strains. In comparison, the stem bark EtOAc and n-BuOH fractions were less potent towards all the MRSA strains, except against the clinical isolate MRSA2 (MIC 0.63 mg/mL; MBC 1.25 mg/mL).

Overall, all the extracts and fractions showed different levels of antibacterial activity against the tested MRSA strains. This variation could be due to the different potencies of the bioactive compounds present in the extracts and fractions leading to different bacteriostatic and bactericidal effects on the bacterial strains, as reported by Qaralleh [15] and Oliveira et al. [16]. Several studies investigated the efficacy of plant extracts and their effective compounds as antibacterial agents to control infections by MRSA, suggesting that the bioactive component(s) of the plant extracts interact with enzymes and proteins of the bacterial cell membrane, causing its disruption, to disperse a flux of protons towards the cell exterior, which induces cell death or may inhibit enzymes necessary for the biosynthesis of amino acids [17].

Besides MIC and MBC values, the antibacterial activity against the MRSA strains was also determined based on the total activity of the extracts and fractions. Total activity is defined as the volume to which the biologically active component (extracts, fractions or compounds) present in 1 g of dried plant material can be diluted and still kill the bacteria [18]. Total activity is useful for the selection of sample material for isolating bioactive compounds. Extracts or fractions with large total activity values are considered the best material for isolating potentially bioactive compounds. As shown in Table 3, the total activity values of the extracts and fractions of *A. pavarii* leaf and stem bark demonstrated high variation. The leaf methanolic extract diluted in 4007.60 mL of solvent can still inhibit the growth of MRSA ATCC 700699 (total activity: 4007.60 mL/g). The leaf n-BuOH fraction possessed higher total activity against MRSA ATCC 700699, with a value of 2235.89 mL/g. The leaf EtOAc fraction has higher total activity against MRSA2 and MRSA ATCC 700699, with 2158.97 and 1078.10 mL/g values, respectively. Both the extract and fractions of the stem bark exhibited lower total activity against all the tested MRSA strains as compared to leaf, with values ranging from 54.22 to 452.35.

### 2.3. Time–Kill Curve for Ethyl Acetate Fraction of the Leaf

**Figure 1:**
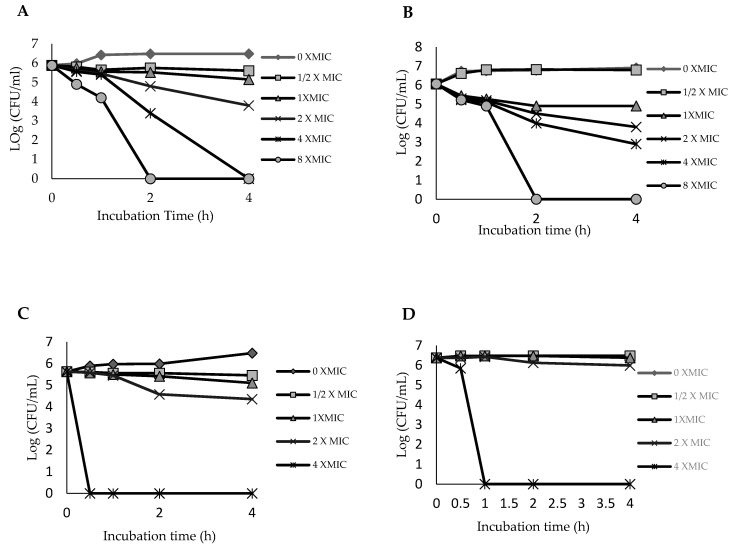
Time–kill curves for leaf EtOAc fraction against (**A**) MRSA ATCC 700699, (**B**) MRSA KCCM 12225, (**C**) MRSA1 and (**D**) MRSA2.

A time–kill assay, using the four bacterial strains, was performed for the leaf EtOAc fraction since it exhibited a stronger antibacterial activity in comparison to the other fractions. Although MIC value gives a good indication of the efficacy of an antimicrobial agent, it provides limited information on the kinetics of the antimicrobial action [19]. A better method of assessing the bactericidal or bacteriostatic activity of an antimicrobial agent over time is by using time–kill kinetics assay, where the effect of various concentrations of the antimicrobial agent over time in relation to the growth stages of the bacteria is monitored [20]. The bacterial strains were thus exposed to the EtOAc fraction, at test concentrations of 0, 0.5, 1, 2, 4 and 8 × MIC over a period of 4 h, and the time–kill curve was plotted. The assay results for MRSA ATCC 700699 (Figure 1A) revealed that the bacteria were completely killed after 4 h when a concentration of 4 × MIC (0.63 mg/mL) was used and after 2 h with the higher concentration of 8 × MIC (1.25 mg/mL). In terms of practical application, the 4 h killing time would be more preferred since the effect was obtained using a lower concentration (0.63 mg/mL) of the disinfecting agent. This condition is similar to that of a drug that exhibits a concentration-dependent bactericidal action, where the bactericidal effect is dependent on the dose of the leaf EtOAc fraction rather than on incubation time [21]. On the other hand, the time–kill curves for MRSA KCCM 12255 (Figure 1B) showed that the time–kill endpoint was achieved after 2 h incubation with a higher concentration of 8 × MIC (2.5 mg/mL). Meanwhile, in the case of the clinical isolates, as illustrated in Figure 1C,D, the time–kill endpoint could only be achieved with a concentration of 4 × MIC (5 mg/mL) after 1 h of incubation.

The data demonstrated that the bactericidal ability of the leaf EtOAc fraction is dependent on concentration and the bacterial strain. Generally, the time–kill kinetics results reasserted the expectation that a more concentrated sample will kill the microorganism in a shorter period of time. An increase in concentrations of plant extracts leads to an increase in the diffusion of phytochemicals into the cell membrane of bacteria, thus causing membrane destruction [22]. Furthermore, the bioactive compounds in the fraction may inhibit the synthesis of essential metabolites such as folic acid by preventing the enzymatic reaction. The protein synthesis in the microorganisms also can be inhibited if the bioactive compounds interfere and change the shape of the ribosome, which may lead to misreading of genetic code on mRNA [22]. The results of this time–kill kinetics study, together with the other results presented earlier, including disc diffusion assay, MIC, MBC and total activity determinations, reveal that the *A. pavarii* leaf possesses bacteriostatic and bactericidal effects against the tested MRSA strains, and the bioactive constituents could be largely present in the ethyl acetate fraction. Consequently, the EtOAc fraction was subjected to dereplication using UHPLC-MS/MS in order to gain an insight into the potential bioactive constituents.

### 2.4. UHPLC-ESI–MS/MS Profile of the EtOAc fraction

**Figure 2:**
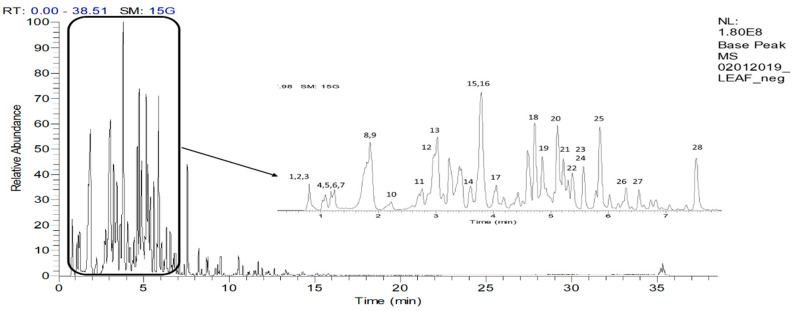
UHPL-ESI-MS/MS base peak chromatogram of the leaf EtOAc fraction of *A. pavarii* in negative ion mode.

Several compounds from the classes of hydroxyquinone (arbutin), phenolic acid (caffeic, ferulic, gallic, rosmarinic, chlorogenic and salicylic acids), flavonoid (catechin, quercetin, dihydroquercetin, isoquercitrin, kaempferol, myricetin, rutin, naringin, neodiosmin, naringenin-7-*O*-glucoside, isovitexin-7-*O*-glucoside and delphinidin-3-*O*-rutinoside) and triterpenoid (oleanolic acid, lupeol and α-amyrin) have been previously reported to be present in *A. pavarii* [11,23,24]. In the present study, 28 compounds were putatively identified from the negative UHPLC-MS/MS spectrum of the leaf EtOAc fraction. The base peak chromatogram is shown in Figure 2, and compounds identified along with their spectral data are shown in Table 4. The results showed that the fraction was rich in phenolic compounds.

#### 2.4.1. Identification of Phenolic Acids and Derivatives

Compounds **2**, **4**, **5**, **6**, **7**, **8**, **11**, **14** and **16** were identified as gallic acid and its derivatives based on the presence of the aglycone fragment ion at *m*/*z* 169 and the characteristic fragment ions at *m*/*z* 271 and 211 in their MS/MS spectra [25]. Compound **5**, with a pseudomolecular ion at *m*/*z* 169.0131, was assigned as gallic acid, showing the characteristic base peak at *m*/*z* 125 for [M-H-CO_2_]^−^. Compounds **2**, **4** and **6**, eluting at three different retention times (0.78, 1.04 and 1.18 min, respectively), were identified as isomers of gallic acid hexoside (I-III). These compounds exhibited pseudomolecular ions at *m*/*z* 331.0668, 331.0669 and 331.0668, respectively, and all three produced a fragment ion at *m*/*z* 169 for [M-H-162]^−^, due to the neutral loss of a hexoxyl moiety. This agrees with previous reports by Mendes et al. [26] and Abu-Reidah et al. [27]. Meanwhile, compound **7** exhibited a pseudomolecular ion at *m*/*z* 343.0668. The compound was assigned as galloylquinic acid based on the presence of base peak at *m*/*z* 169 and fragment ion at *m*/*z* 125 for a further loss of CO_2_, all of which were characteristic fragment ions of gallic acid moiety [28]. Compounds **11** and **16** were identified as *di*-*O*-galloylhexose and *tri*-*O*-galloylhexose, respectively, based on similar fragmentation pattern showing losses of the corresponding number of galloyl moieties and the presence of a base peak at *m*/*z* 169 for the gallic acid aglycone.

Compound **8** has a pseudomolecular ion of *m*/*z* 315.0720, indicative of the molecular formula C_13_H_16_O_9_. It was identified as dihydroxybenzoic acid *O*-hexoside based on fragment ion at *m*/*z* 153 for [M-H-162]^−^, due to the loss of a hexoxyl moiety, and fragment ion at *m*/*z* 109 for [M-H-162-44]^−^ indicating a further loss of CO_2_ moiety, in agreement with Karar and Kuhnert [29]. Meanwhile, compound **14**, which exhibited a pseudomolecular ion of *m*/*z* 329.0878 and base peak at *m*/*z* 167 for [M-H-162]^−^ for a neural loss of a hexoxyl moiety, was assigned as vanillic acid-*O*-hexoside. The assignment was supported by comparison with the fragmentation pattern previously reported by Morales-Soto et al. [30].

#### 2.4.2. Identification of Flavan-3-ol and Derivatives

Compounds **9**, **10**, **12**, **13**, **15**, **17**, **20** and **21** were identified as (epi)catechin and its derivatives based on the presence of fragment ions at *m*/*z* 289 and 125, corresponding to the (epi)catechin aglycone [31]. Compound **9**, which displayed a pseudomolecular ion at *m*/*z* 305.0663, was identified as (epi)gallocatechin based on the fragment ion at *m*/*z* 179 for [M-H-126]^−^, due to the characteristic loss of the trihydroxybenzene moiety [32]. Compound **10**, with pseudomolecular ion at *m*/*z* 451.1254, was identified as (epi)catechin-3-*O*-hexoside based on the fragment ion at *m*/*z* 289, for the loss of a hexoxyl moiety [33]. Compounds **13** (Rt = 5.44 min) and **15** (Rt 7.74 min) showed similar pseudomolecular ions at *m*/*z* 289.0714 and 289.0717, respectively. By comparison of their elution order with a previous study by Stöggl et al. [34], the compound eluted earlier was identified as catechin while the one eluted later was identified as epicatechin. Both compounds yielded the fragment ions at *m*/*z* 137 and 151 which were the results of retro-Diels–Alder (RDA) cleavage at ring C of the flavan-3-ol structure. 

Three compounds (**12**, **17** and **21**) were identified as the dimeric forms of B-type proanthocyanidins (PAs), which could be differentiated from the A-type Pas with the extra 2 Da in their pseudomolecular ion [35]. Compound **12**, showing pseudomolecular ion at *m*/*z* 577.1334, was identified as the (epi)catechin + (epi)catechin. The compound also exhibited a fragment ion at *m*/*z* 425 ([M–H-152]^−^), which was due to the characteristic RDA cleavage at ring C of the dimer top unit [35]. Another fragment ion at *m*/*z* 407 ([M-H-152-18]^−^) due to the subsequent loss of a water molecule from the parent molecule was also observed. The presence of two other dimeric derivatives, **17** and **21**, was also indicated by the pseudomolecular ions at *m*/*z* 729.1458 (Rt = 4.03 min) and 729.1453 (Rt = 5.25 min). These compounds were identified as (epi)catechin gallate + (epi)catechin isomers based on the fragment ion at *m*/z 577 indicative of galloyl moiety losses ([M-H-152]^−^) from the parent ion [36]. Compound **20** at Rt = 5.20 min was identified as (epi)catechin-3-*O*-gallate. It displayed a pseudomolecular ion at *m/z* 441.0823. Its fragmentation pattern showed a fragment ion at *m*/*z* 289 for [M-H-169]^−^, which corresponded to a loss of gallic acid moiety via cleavage of the ester bond and loss of the (epi)catechin unit [37].

#### 2.4.3. Identification of Flavonols and Derivatives

The ethyl acetate fraction also contained the flavonol quercetin (**28**) and several of its derivatives (**18**, **19**, **23**, **25**, **26**, **27** and **28)**. Quercetin (**28**) was identified based on its pseudomolecular ion at *m*/*z* 301.03 and fragment ions at *m*/*z* 271, 255, 179 and 151 [36]. Compound **18**, with pseudomolecular ion at *m*/*z* 615.0997, displayed fragment ions at *m*/*z* 463 for [M-H-169]^−^, indicating loss of a galloyl moiety, and at *m*/*z* 301 for [M-H-331]^−^, indicating an additional loss of a hexoxyl moiety. Compound **18** was thus deduced to be quercetin-*O*-galloylhexoside, based on these data and data reported by Mendes et al. [26].

Compounds **19**, **23**, **25** and **26** were assigned as quercetin-3-*O*-deoxyhexosylhexoside, quercetin-3-*O*-pentoside, quercetin-3-*O*-deoxyhexoside and quercetin-3-*O*-hexoside. These compounds exhibited pseudomolecular ions at *m*/*z* 609.1463, 433.0775, 447.0931 and 463.0885, respectively. The transition of these ions to the aglycone ion (Y_0_^−^) at *m*/*z* 301 revealed the losses of the respective sugar moieties [34]. The glycosylation at the C-3 position of these compounds was determined by the higher relative abundance of their radical aglycone ion ([Y_0_ H]^−^ *m*/*z* 300) than the Y_0_^−^ ion (*m*/*z* 301) [38]. Compound **27**, with a pseudomolecular ion at *m*/*z* 583.1099, was assigned as quercetin-*O*-(*p*-hydroxy)benzonylhexoside. The compound showed a fragment ions at *m*/*z* 463 for [M-H-120]^−^, indicating a loss of hydroxybenzoyl moiety, and *m*/*z* 301 ([M-H-282]^−^) for a further loss of hexoxyl moiety, in agreement with data reported by Jaiswal et al. [36].

Compound **22** was identified as myricetin-3-*O*-hexoside based on the presence of fragment ions at *m*/*z* 317, 316, 179 and 151, corresponding to the aglycone myricetin. The deprotonated aglycone peak observed at *m*/*z* 316.02 [M-H-162]^−^ was due to the loss of a hexoxyl moiety [39]. Compound **24** with a pseudomolecular ion at *m*/*z* 447.093 was identified as kaempferol-3-*O*-hexoside. This compound showed characteristic fragment ion at *m*/*z* 285 due to the loss of sugar moiety and fragment ions at *m*/*z* 255 and 227 which are due to the loss of [M-162-CHO]^−^ and [M-162-H_2_O-CO], respectively. Similarly, the fragment ions at *m*/*z* 179 and 151 were due to RDA cleavage of C-ring [39]. Attachment of the sugar moiety at the C-3 position of these compounds was also determined based on the relative abundance of [Y_0_ H]^−^ and Y_0_^−^ ions [38].

#### 2.4.4. Identification of Other Compounds

Compound **1**, with a pseudomolecular ion at *m*/*z* 191.0555 (C_7_H_12_O_6_), was identified as quinic acid. It yielded fragment ions at *m*/*z* 171 ([M-H-H_2_O]^−^), 127.04 ([M–H-CO_2_-H_2_O]^−^) and 109 ([M–H-CO_2_-2H_2_O]^−^) [28]. Compound **3**, with pseudomolecular ion at *m*/*z* 271.0453 (C_12_H_16_O_7_), was identified as arbutin; it yielded a fragment ion at *m*/*z* 108 for [M-H-162]^−^ due to loss of the hexose moiety [40].

The UHPLC-MS/MS results showed that the flavonoids and phenolic acid components are major secondary metabolites in the ethyl acetate fraction of *A. pavarii* leaf. Furthermore, among the identified compounds, several of them have been previously reported to possess antibacterial activity against MRSA. Shibata et al. [41] reported that gallic acid has antibacterial activity against MRSA with MIC value of 62.5 μg/mL. Catechins are often linked to antimicrobial effects associated with their interactions with the microbial cell membrane [42]. Cushnie et al. [43] reported that membrane disruption by catechins causes potassium leakage in MRSA strain, which is the first indication of membrane damage in microorganisms [44]. In addition, several studies have shown that the effectiveness of β-lactams can be enhanced by combining them with epigallocatechin gallate [45,46] and epicatechin gallate [47]. Meanwhile, Su et al. [48] reported that quercetin exhibited inhibitory effect against different MRSA strains, with MIC values ranging from 31.25 to 125 μg/mL, while rutin, a quercetin-3-*O*-deoxyhexosylhexoside, was reported to inhibit MRSA with MIC value of 250 μg/mL [49]. Besides, arbutin was reported to exert antibacterial activity against MRSA with MIC value of 10 mg/mL and MBC value of 20 mg/mL [50]. Therefore, the presence of these compounds, especially the flavonoids and phenolic acids, could have contributed significantly to the antibacterial activity of the leaf ethyl acetate fraction of *A. pavarii*.

## 3. Materials and Methods

### 3.1. Plant Materials, Extraction and Fractionation

The leaf and stem bark of *A. pavarii* were obtained from Al Jabal Al Akhdar region, Northeast Libya in March 2016 and identified by Dr. Abdulamid Alzerbi, a botanist at Biology Department of Benghazi University, Libya. The leaf and stem bark were dried under shade before being pulverized into a powder using a mechanical grinder (model: MX1100XT11CE, Waring, S/NoB 8643, Atlanta, GA, USA). The powdered plant material was sieved with a steel sieve (80 mesh) to obtain a uniform fine powder. For extraction, 1500 g of the ground leaf and 500 g of the stem bark were separately mixed with methanol at 1:10 solid-to-liquid ratio. The mixtures were sonicated at 35 °C for 60 min with a frequency of 53 kHz using an ultrasonic water bath (Branson, model 8510E-MTH, Danbury, CT, USA). The crude methanolic extracts were filtered (Whatman No. 1 filter paper, USA), and the collected filtrate was concentrated at 45 °C under reduced pressure using a rotary evaporator (Buchi, USA). The crude methanolic extracts were further fractionated using liquid–liquid fractionation to obtain solvent fractions of different polarities, namely hexane, chloroform, ethyl acetate (EtOAc) and n-butanol (n-BuOH) fractions (Merck, Darmstadt, Germany). The yields and physical appearance of the various extracts and fractions are tabulated in Table 5.

### 3.2. Bacterial Strains and Preparation of Inoculum

MRSA ATCC 700699 was obtained from the American Type Culture Collection (Rockville, MD, USA) while MRSA KCCM 12255 was obtained from the Korean Culture Center of Microorganisms (Seoul, South Korea). Two clinical isolates (MRSA1 and MRSA2) were collected from the nasal swab of a 4th-year medical student from University Putra Malaysia, Malaysia. The MRSA strains are kept at the Laboratory of Natural Products (Institute of Bioscience, UPM, Malaysia). The MRSA ATCC 700699, MRSA KCCM 12255 MRSA1 and MRSA2 were grown on Mueller Hinton agar (MHA) (Difco, Franklin Lakes, NJ, USA) aerobically for 24 h at 37 °C, whereas inoculum cell suspension was prepared by transferring and incubating a single colony of each bacterial species in 10 mL of Mueller Hinton broth (MHB) at 37 °C overnight with 200 rpm agitation. Then, 1 µL of bacteria suspension was transferred to new MHB in a ratio of 1:10 to yield an inoculum size of 10^6^ CFU/mL.

### 3.3. Disc Diffusion Assay

Antibacterial activity was evaluated using agar diffusion assay, according to Rukayadi et al. [51]. Briefly, an inoculum of the bacterial strain was streaked on the surface of MHA plates using a sterile cotton swab. Sterile 6 mm filter paper discs (Whatman, Germany) were prewetted with 10 µL aliquot of the test extracts or fractions, prepared in DMSO at a concentration of 10 mg/mL. The discs were then placed on the inoculated plates at an appropriate distance from each other. Positive (chlorhexidine, 0.1% CHX, St Louis, MO, USA) and negative (dimethyl sulfoxide, 10% DMSO, Merck, Darmstadt, Germany) control discs were similarly prepared and placed on each test plate. Inoculated plates were subsequently incubated for 24 h at 37 °C and observed for inhibition zones. All experiments were conducted in triplicate, and inhibition zone diameter (IZD) was measured in mm. Antibacterial activity was expressed as the percentage of relative inhibition zone diameter (RIZD) with respect to standard antibiotic (0.1% CHX), according to Alsohaili and Al-fawwaz [52], and calculated using the following formula:

% RIZD = [(IZD_sample_−IZD_negative control_)/IZD_standard antibiotic_] × 100 (1)

### 3.4. Determination of Minimum Inhibitory Concentration (MIC) and Minimum Bactericidal Concentration (MBC) Values

The MIC and MBC values of the test samples against the MRSA strains were established as described by the Clinical and Laboratory Standards Institute (CLSI) [19]. The determination was performed in a 96-well round-bottom microtiter plate (Greiner, Germany) using a 2-fold standard broth microdilution method with an inoculum of about 10^6^ CFU/mL. The first well, designated as the negative control, was filled with 100 µL MHB. The second well, designated as the positive control, was filled with 100 µL of the bacterial suspension. A 100 µL aliquot of the test extract or fraction, prepared at a concentration of 10 mg/mL, was then added to the 12th well. Two-fold dilutions were then made from the 12th well down to the 3rd well. Therefore, the 12th well contained the highest concentration (5 mg/mL), while the 3rd well contained the lowest concentration (0.01 mg/mL). The plate was then incubated aerobically for 24 h at 37 °C. After the incubation period, the MIC of the test extract or solvent fraction was determined. The MIC value is defined as the lowest concentration of the test sample that inhibited bacterial growth completely. For determining the MBC value, a 10 µL aliquot of the suspension in each of the 12 wells of the MIC determination was subcultured on an MHA plate. The plate was then incubated for 24 h, at 37 °C. After the incubation period, the plate was observed for bacterial growth and the MBC value was determined. The MBC is defined as the lowest concentration of the test sample that killed the bacterial strain completely. The MIC and MBC values were determined in duplicate. Chlorhexidine (0.1% CHX, St Louis, MO, USA) was used as a positive control. The antimicrobial activity of plant extracts may be expressed in different ways, including total activity values [18]. The total activity of the extract and the fractions was estimated as follows:

Total activity = Quantity of material extracted from 1g of plant material/MIC(2)

### 3.5. Time–Kill Curve

Time–kill assay against the MRSA strains was performed according to Ramli et al. with slight modifications [22]. Briefly, the inoculum suspension of MRSA was diluted to approximately 10^6^ CFU/mL. The ethyl acetate fraction of the leaf was diluted with the MHB medium containing inoculum to obtain final concentrations of 0 × MIC, 0.5 × MIC, 1 × MIC, 2 × MIC, 4 × MIC and 8 × MIC for MRSA ATCC 700699 and MRSA KCCM 12255 and final concentrations of 0 × MIC, 0.5 × MIC, 1 × MIC, 2 × MIC and 4 × MIC for MRSA1 and MRSA2. Cultures (1 mL final volume) were incubated at 30 °C with 200 rpm agitation. At predetermined time points (0, 0.5, 1, 2 and 4 h), 10 µL aliquots were transferred to clean microcentrifuge tubes. The aliquots were serially diluted with 990 µL of 1% phosphate-buffered saline (PBS), and 20 µL was staked onto the MHA plates. The number of colonies formed on the plates after incubation at 30 °C for 24 h was counted and the number of CFU/mL was calculated. Assays were carried out in triplicate. The graph of log CFU/mL versus time was plotted as described by Ramli et al. [22].

### 3.6. UHPLC-ESI-MS/MS Analysis

The bioactive fraction was separated using a Hypersil Gold C18 reversed-phase column (2.1 × 100 mm, 1.9 µm, Thermo, USA) on a Thermos Scientific Ultimate 3000 (Bremen, Germany) with a mobile phase consisting of LCMS grade water (solvent A) and acetonitrile (solvent B), each containing 0.1% formic acid flowing at 0.4 mL/min. The programmed gradient system consisted of 0 min (95% A), 1 min (95% A), 20 min (5% A), 25 min (5% A), 25.1 min (95% A) and 35 min (95% A). The sample of 1 mg/mL (*w*/*v*) was prepared by dissolving 1 mg of a dried sample of the active fraction with 1 mL of methanol. The resultant mixture was then filtered using 0.22 μm Nylon membranes, and then 10 µL of the filtrate was auto-injected. The MS analysis was done on a Q-Exactive Focus Orbitrap LC-MS/MS system. The ESI-MS parameters were set as follows: negative mode, collision energy of 3.5 kV, capillary temperature 350 °C, auxiliary gas heater temperature 0 °C, sheath gas flow rate 40 arbitrary units and auxiliary nitrogen gas (99% pure) flow rate 8 arbitrary units. Then, the mass resolution was set to 70,000 full width at half maximum (FWHM) and a full scan of 150–2000 amu. The identification analysis was carried out by comparing the obtained MS/MS data with the literature.

### 3.7. Data Analysis

Microsoft Excel (Version 2010) was employed to perform the statistical analysis. Disc diffusion results were given as a mean ± standard deviation with three replicates.

## 4. Conclusions

In this study, the leaf and stem bark of *A. pavarii* were evaluated for anti-MRSA activity. The antibacterial activity was performed using disc diffusion agar test, MIC and MBC assays, in which the methanolic extracts and fractions of leaf and stem bark of *A. pavarii* demonstrated potential antibacterial activity against the tested MRSA strains. Among the extracts and fractions, the EtOAc fraction of *A. pavarii* leaf revealed the highest antibacterial activity against all tested MRSA strains, with activity ranging from moderate to significant (MIC 0.08–1.25 mg/mL). In time–kill analysis, the MRSA strains were found to be completely killed after exposure to this fraction for 30 min to 2 h at 4× MIC and 8× MIC, revealing a remarkable capacity to inhibit or kill the MRSA strains. The UHPLC-ESI-MS/MS profiling of the bioactive fraction revealed that it contains high amounts of polyphenolic compounds. Phenolic acid and flavonoids were the main components and could be responsible for the bioactivity. The present findings add support for the traditional medicinal use of *A. pavarii* and highlight its potential as a source of natural antibacterial agents for future exploitation as natural antibiotics in the fight against MRSA prevalence.

## Figures and Tables

**Table 1:** Inhibition zones of leaf and stem bark crude methanolic extracts and solvent fractions of *A. pavarii* against methicillin-resistant *Staphylococcus aureus* (MRSA) strains.

MRSA Strains	CHX	CH_3_OH		EtOAc		n-BuOH	
		IZD	% RIZD	IZD	% RIZD	IZD	% RIZD
		Leaf					
**ATCC 700699**	8.00 ± 0.00	9.33 ± 0.57	120.83 ± 7.22	13.67 ± 0.57	170.83 ± 7.22	8.33 ± 0.57	100.00 ± 5.59
**KCCM 12255**	7.00 ± 0.00	8.00 ± 0.00	114.29 ± 0.00	12.00 ± 0.00	171.43 ± 0.00	7.00 ± 0.00	100.00 ± 0.00
**MRSA1**	10.33 ± 0.57	9.67 ± 0.57	93.58 ± 5.59	13.67 ± 1.15	132.30 ± 5.59	n.a	n.d
**MRSA2**	10.33 ± 0.57	9.33 ± 0.57	83.90 ± 5.59	13.00 ± 0.00	125.85 ± 0.00	n.a	n.d
		**Stem bark**					
**ATCC 700699**	8.00 ± 0.00	10.00 ± 0.00	125.00 ± 0.00	9.00 ± 0.00	112.50 ± 0.00	9.00 ± 0.00	112.50 ± 0.00
**KCCM 12255**	7.00 ± 0.00	7.00 ± 0.00	100.00 ± 0.00	8.00 ± 0.00	114.29 ± 0.00	7.00 ± 0.00	100.00 ± 0.00
**MRSA1**	10.33 ± 0.57	9.00 ± 0.00	87.12 ± 0.00	9.00 ± 0.00	87.12 ± 0.00	n.a	n.d
**MRSA2**	10.33 ± 0.57	7.67 ± 0.57	74.22 ± 5.59	8.33 ± 0.57	80.67 ± 5.59	7.33 ± 0.57	70.99 ± 5.59

CH_3_OH = methanol extract, EtOAc = ethyl acetate fraction, n-BuOH = butanol fraction. Hexane and chloroform fractions showed no inhibition zones. MRSA1 and MRSA2 are clinical isolates, n.a = no activity (no inhibition zone detected). n.d = not detected. Diameter of inhibition zones in mm (including disc). Positive control: 0.1% CHX; negative control: 10% DMSO. Values are expressed as means ± standard deviation (SD).

**Table 2:** Minimum inhibitory concentration (MIC) and minimum bactericidal concentration (MBC) values (mg/mL) of leaf and stem bark crude methanolic extracts and solvent fractions of *A. pavarii* against MRSA strains.

MRSA Strains	Parts	CH_3_OH		EtOAc		n-BuOH		CHX	
		MIC	MBC	MIC	MBC	MIC	MBC	MIC	MBC
ATCC 700699	Leaf	0.08	0.16	0.08	0.16	0.04	0.08	0.02	0.63
	Stem bark	0.63	1.25	1.25	2.50	2.50	5.00		
KCCM 12255	Leaf	0.63	1.25	0.31	1.25	0.63	1.25	0.02	0.63
	Stem bark	1.25	2.50	1.25	2.50	2.50	5.00		
MRSA1	Leaf	1.25	2.50	1.25	2.50	2.50	5.00	0.03	0.13
	Stem bark	1.25	2.50	1.25	2.50	2.50	5.00		
MRSA2	Leaf	1.25	2.50	1.25	2.50	1.25	2.50	0.03	0.13
	Stem bark	1.25	2.50	0.63	1.25	0.63	1.25		

CH_3_OH = methanol extract, EtOAc = ethyl acetate fraction, n-BuOH = butanol fraction, CHX = 0.1% chlorhexidine (standard antibiotic). Hexane and chloroform fractions showed no inhibition zones. MRSA1 and MRSA2 are clinical isolates.

**Table 3:** Total activity of leaf and stem bark crude methanolic extracts and solvent fractions of *A. pavarii* against MRSA strains.

MRSA Strains	Total Activity in (mL/g)					
	Leaf			Stem Bark		
	CH_3_OH	EtOAc	n-BuOH	CH_3_OH	EtOAc	n-BuOH
ATCC 700699	4007.6	1078.1	2235.89	452.35	61.18	54.22
KCCM 12255	500.8	269	139.52	226.16	61.18	54.22
MRSA1	250.4	67.36	34.88	113.08	61.18	54.22
MRSA2	62.6	2158.97	69.76	226.16	122.37	216.90

**Table 4:** Compounds identified in the leaf EtOAc fraction of *A. pavarii*.

No	Retention Time (Rt) (min)	[M-H]^−^ (*m*/*z*)	MS/MS Fragment Ions (*m*/*z*)	Compound Identity	Molecular Formula
**Phenolic Acids and Derivatives**					
2	0.78	331.0668	271.05, 211.02, 169.01	Gallic acid hexoside I	C_13_H_16_O_10_
4	1.04	331.0669	271.05, 211.02, 169.01	Gallic acid hexoside II	C_13_H_16_O_10_
5	1.18	169.0131	125.02	Gallic acid	C_7_H_6_O_5_
6	1.22	331.0668	271.05, 211.02, 169.01	Gallic acid hexoside III	C_13_H_16_O_10_
7	1.23	343.0668	191.06, 169.01, 125.02	Galloylquinic acid	C_14_H_16_O_10_
8	1.73	315.0720	153.02, 152.01, 109.03, 108.02	Dihydroxybenzoic acid-*O*-hexoside	C_13_H_16_O_9_
11	2.89	483.0774	439.09, 424.54, 331.07, 313.06, 287.08, 271.05, 211.02, 169.01	*Di*-*O*-galloylhexose	C_20_H_20_O_14_
14	3.43	329.0878	167.03, 152.01, 123.04, 108.02	Vanillic acid-*O*-hexoside	C_14_H_18_O_9_
16	3.95	635.0888	465.07, 313.06, 271.05, 211.02, 169.01	*Tri*-*O*-galloylhexose	C_27_H_24_O_18_
**Flavan-3-ol and Derivatives**					
9	1.83	305.06638	261.08, 179.03, 138.03, 137.02, 125.02	(Epi)gallocatechin	C_15_H_14_O_7_
10	2.11	451.1254	289.07, 245.08, 151.04, 125.02	(Epi)catechin-3-*O*-hexoside	C_21_H_24_O_11_
12	2.90	577.1334	451.10, 425.09, 407.08, 289.07, 287.06, 245.08, 125.02	(Epi)catechin +(epi)catechin I	C_30_H_26_O_12_
13	3.11	289.0714	271.06, 245.08, 179.03, 165.02, 150.03, 137.02, 125.02	Catechin	C_15_H_14_O_6_
15	3.88	289.0717	271.06, 245.08, 179.03, 165.02, 150.03, 137.02, 125.02	Epicatechin	C_15_H_14_O_6_
17	4.03	729.1458	577.14, 559.13, 451.10, 425.09, 407.08, 289.07, 125.02	(Epi)catechin gallate + (epi)catechin I	C_37_H_30_O_16_
20	5.20	441.0823	289.07, 245.08, 203.07, 169.01	(Epi)catecin gallate	C_22_H_18_O_10_
21	5.25	729.1453	577.11, 407.08, 425.09, 289.07, 125.02	(Epi)catechin gallate + (epi)catechin II	C_37_H_30_O_16_
**Flavonols and Derivatives**					
18	4.72	615.0989	463.09, 300.03, 301.03, 271.02, 179.00, 151.00, 169.01	Quercetin-*O*-galloylhexoside	C_28_H_24_O_16_
19	4.98	609.1463	301.03, 300.03, 271.02, 255.03	Quercetin-3-*O*-deoxyhexosyl-hexoside	C_27_H_30_O_16_
22	5.40	463.0884	317.03, 316.02, 287.02, 271.02, 179.00, 151.00	Myricetin-3-*O*-deoxyhexoside	C_21_H_20_O_12_
23	5.55	433.0775	301.03, 300.03, 271.02, 255.03	Quercetin-3-*O*-pentoside	C_20_H_18_O_11_
24	5.56	447.0931	285.04, 284.03, 255.03, 227.03	kaempferol-3-*O*-hexoside	C_2120_O_11_
25	5.99	447.0931	301.03, 300.03, 271.02, 255.03	Quercetin-3-*O*-deoxyhexoside	C_21_H_20_O_11_
26	6.30	463.0885	301.03, 300.03, 271.07, 255.03	Quercetin-3-*O*-hexoside	C_21_H_20_O_12_
27	6.42	583.1099	463.09, 301.03, 300.03, 271.03, 255.03	Quercetin-*O*-(*p*-hydroxy) benzonylhexoside	C_28_H_24_O_14_
28	7.53	301.0354	271.02, 255.03, 179.00, 151.00, 149.02, 121.03, 121.03, 107.01	Quercetin	C_15_H_10_O_7_
**Others**					
1	0.76	191.0555	171.03, 127.04, 109.03, 93.03	Quinic acid	C_7_H_12_O_6_
3	0.80	271.0453	211.02, 108.02	Arbutin	C_12_H_16_O_7_

**Table 5:** Yields of extracts and solvent fractions of *Arbutus pavarii*.

Plant Part	Solvent	Weight (g)	Yield %	Physical Appearance
**Leaf**	CH_3_OH	470.00	31.13	Dark greenish brown gum
	Hex	18.60	3.95	Dark green gum
	CHCl_3_	24.90	5.29	Green gum
	EtOAc	126.48	26.91	Dark orange gum
	*n*-BuOH	131.00	27.87	Brown gum
**Stem Bark**	CH_3_OH	141.35	28.27	Greenish brown gum
	Hex	11.42	8.08	Dark green gum
	CHCl_3_	3.65	2.58	Green gum
	EtOAc	38.24	27.05	Dark brown gum
	*n*-BuOH	67.78	47.95	Dark brown gum
